# A novel rat model of stable posttraumatic joint stiffness of the knee

**DOI:** 10.1186/s13018-018-0894-y

**Published:** 2018-07-25

**Authors:** Andreas Baranowski, Ludwig Schlemmer, Katharina Förster, Stefan G. Mattyasovszky, Ulrike Ritz, Daniel Wagner, Pol M. Rommens, Alexander Hofmann

**Affiliations:** 1 Department of Orthopaedics and Traumatology, University Medical Center, Johannes Gutenberg University, Langenbeckstraße 1, 55131 Mainz, Germany; 2 Department of Traumatology and Orthopaedics 1, Westpfalz-Medical Centre Kaiserslautern, Hellmut-Hartert-Str 1, 67655 Kaiserslautern, Germany

**Keywords:** Small animal model, Posttraumatic joint stiffness, Myofibroblasts, Contracture development

## Abstract

**Background:**

Animal models of posttraumatic joint stiffness (PTJS) are helpful in understanding underlying mechanisms, which is important for developing specific treatments and prophylactic therapies. Existing rat models of PTJS in the knee failed to show that the created contracture does not resolve through subsequent remobilization. Our objective was to establish a rat model of persisting PTJS of the knee and compare it to existing models.

**Methods:**

Thirty skeletally immature male Sprague Dawley rats underwent surgical intervention with knee hyperextension, extracartilaginous femoral condyle defect, and Kirschner (K)-wire transfixation for 4 weeks with the knee joint in 146.7° ± 7.7° of flexion (*n* = 10 per group, groups I–III). After K-wire removal, group I underwent joint angle measurements and group II and group III were allowed for 4 or 8 weeks of free cage activity, respectively, before joint angles were measured. Eighteen rats (*n* = 6 per group, groups Ic–IIIc) served as untreated control.

**Results:**

Arthrogenic contracture was largest in group I (55.2°). After 4 weeks of remobilization, the contracture decreased to 25.7° in group II (*p* < 0.05 vs. group I), whereas 8 weeks of remobilization did not reduce the contracture significantly (group III, 26.5°, *p* = 0.06 vs. group I). Between 4 and 8 weeks of remobilization, no increase in extension (26.5° in group III, *p* = 0.99 vs. group II) was observed. Interestingly, muscles did not contribute to the development of contracture.

**Conclusion:**

In our new rat model of PTJS of the knee joint, we were able to create a significant joint contracture with an immobilization time of only 4 weeks after trauma. Remobilization of up to 8 weeks alone did not result in full recovery of the range of motion. This model represents a powerful tool for further investigations on prevention and treatment of PTJS. Future studies of our group will use this new model to analyze medical treatment options for PTJS.

## Background

Posttraumatic joint contracture represents a pathological reduction of the range of motion (ROM), which has a devastating impact on the articular function and activities of daily life [1–3]. Injuries of the joint capsule and/or the nearby bone are often associated with development of joint contractures, also called posttraumatic joint stiffness (PTJS). Injuries to both the articular (capsule, ligaments, cartilage, bone, menisci) and extra-articular structures (muscles, tendons, and skin) may contribute to the development and progress of PTJS. Current therapies focusing on prevention and treatment of PTJS include physiotherapy, continuous passive motion, and, in case of failure, surgical treatment [2, 4]. However, PTJS may be very difficult to treat and often persists despite of treatment.

In case of preserved joint surface integrity and congruence, the joint capsule seems to be the major contributor to PTJS [5, 6]. Animal models were developed to investigate the underlying mechanisms of PTJS and mostly focus on the consequences of prolonged immobilization [7–20]. Although some recent studies imply that immobilization alone is sufficient to produce a stable joint contracture [10, 13], these models do not fully reproduce the mechanism of PTJS in humans. Recent studies showed that the factors joint immobilization, injury of soft tissue, and bone must be considered to closely imitate PTJS in humans [21–23]. Furthermore, the development of an animal model of PTJS, which provides stable and reproducible contractures, remains a challenge, because remobilization of the injured joints may lead to a rapid and almost full recovery of all relevant structures, particularly in rats [24, 25]. Therefore, the “stability” of the contracture after remobilization is one of the most critical factors to be addressed in animal models of PTJS.

In this study, we established a reproducible model of stable posttraumatic knee joint flexion contracture in rats. Handling, anatomical sizes, more simple investigation methods, and logistics in this small animal model may be advantageous for the development of treatment and prevention strategies of PTJS in the future.

## Methods

### Study design

Forty-eight skeletally immature male Sprague Dawley rats from Janvier Labs (CS 4105 Le Genest-Saint-Isle, F-53941 Saint-Berthevin Cedex, France) were used. The animals had a mean weight of 408 ± 31 g and an age of 10 weeks at the beginning of the experiment. They were kept at room temperature in a 12-h light/dark cycle in our enclosed laboratory facility with biosafety level 1. All animals were housed individually in Makrolon type IV cages (Zoonlab, Castrop-Rauxel, Germany) with a floor space of 1815 cm^2^ and a height of 20 cm. Free cage activity and access to food and water were allowed ad libitum. This study was approved by the local ethics committee (ID 23 177-07/G 13-1-043 E1). Thirty animals were allocated to the experimental groups (I–III) and 18 animals to the control groups (Ic–IIIc, Table 1). The number of animals was based on sample size calculation based on results of previous studies [6, 21, 22]. Our experimental unit was a single rat. All animals in groups I–III underwent the same surgical procedure and differed in the duration of remobilization. Surgical procedures were carried out in the afternoon and early evening. The order in which the animals in the different experimental groups were treated and assessed was not randomized. We defined the joint angle/extension deficit of the knee joint as the primary experimental outcome parameter.

**Table 1:** Group allocation

Groups	Procedure	Joint immobilization time (in weeks)	Joint remobilization time (in weeks)
I (*n* = 10)	Experimental groups	4	None
II (*n* = 10)		4	4
III (*n* = 10)		4	8
Ic (*n* = 6)	Control groups	None	4
IIc (*n* = 6)		None	8
IIIc (*n* = 6)		None	12

### Anesthesia and surgical procedure

Anesthesia was initiated via inhalation of 1% isoflurane and maintained with a subcutaneous injection of 0.005 mg/kg fentanyl, 4.0 mg/kg midazolam, and 0.375 mg/kg Medetomidin. The animals’ legs were shaved with an electric clipper and prepped with Braunol 7.5%. The leg to be operated on was allocated sequentially: half of the animals in groups I–III received the surgical intervention on the left side, the other half on the right side.

**Fig. 1:**
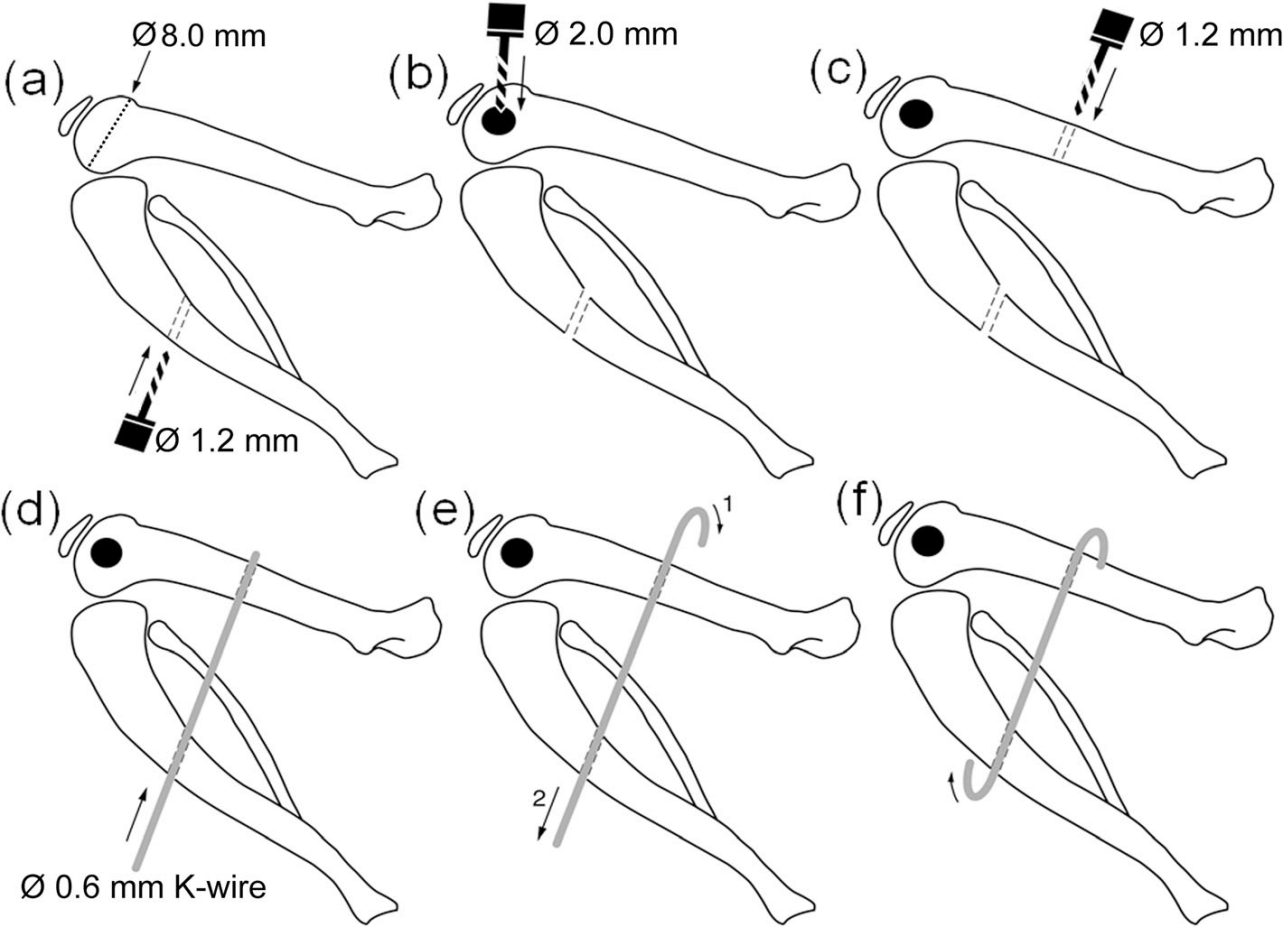
Surgical procedure. **a** Ascending transtibial drilling. **b** Two-millimeter condylar drill hole. **c** Descending transfemoral drilling. **d** Insertion of K-wire. **e** Bending and pulling of K-wire. **f** Fixation in 145° of knee flexion

We used a hyperextension of − 45° of the knee joint in order to disrupt the posterior joint capsule as previously described [24, 25]. Thereafter, accidental fractures or dislocation of the epiphysis were ruled out using plane radiographs in the anteroposterior and lateral projections. Stab incisions of the skin were made over the lateral thigh and the anteromedial tibia. An ascending hole of 1.2-mm diameter was drilled into the tibial diaphysis through the anteromedial tibial incision (Fig. 1a). A lateral approach to the femur was carried out by dissecting the fascia and anterior retraction of the vastus lateralis muscle. The femoral condyle in a 10-week-old male Sprague Dawley rat measures about 8.0 mm at its widest point in the sagittal plane from anterior to posterior. In the coronal plane, the distance from medial to lateral has a length of about 6.0 mm (Fig. 1a). The lateral femoral condyle was exposed and a 2-mm-thick and 4-mm-deep hole was drilled into the non-cartilaginous part to create a standardized intraarticular bony lesion (Fig. 1b). Attention was paid to avoid damage to the lateral collateral ligament. This bony lesion mimics a juxta-articular fracture and causes bleeding into the joint [21, 26, 27]. Another 1.2-mm hole was drilled into the femoral shaft in anteroposterior direction (Fig. 1c). A blunt K-wire of 0.6-mm diameter was driven through the tibial hole, passed the soft tissue posterior to the knee joint, and entered the posterior side of the femoral drill hole (Fig. 1d). The K-wire was bent to form a hook over the femur and pulled back, until a knee flexion of 145° was reached (Fig. 1e). Fixation angle and K-wire position were checked via fluoroscopy. Then, the tibial end of the wire was also bent to insure stable transfixation and cut below the skin level (Fig. 1f).

**Fig. 2:**
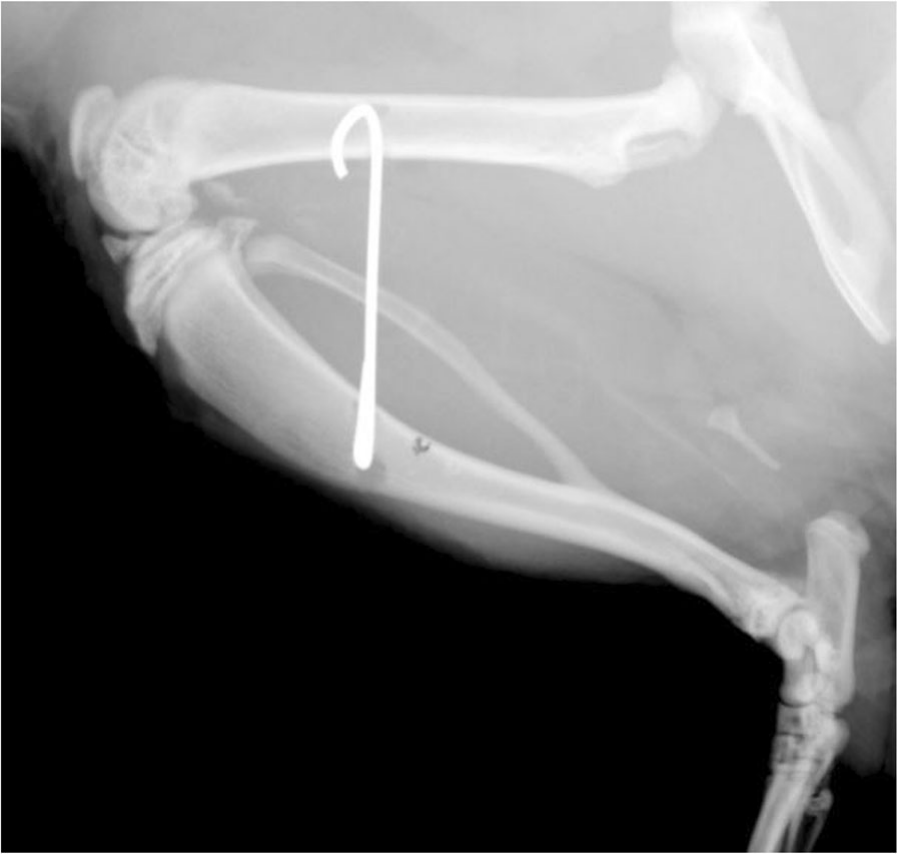
Postoperative lateral X-ray of the knee joint. K-wire immobilization in a 145° flexed knee position

The wounds were carefully rinsed with sterile saline, and proper patellofemoral articulation was assessed. Tibial and femoral fasciae were closed using 4-0 Vicryl® sutures. Finally, the skin was closed with 4-0 Ethilon® sutures. Following operation, the final position of the K-wire was verified using a lateral plain radiograph of the operated leg (Fig. 2). General anesthesia was antagonized with flumazenil 0.2 mg/kg and atipamezole 1 mg/kg. Drinking water was supplemented with tramadol 1 mg/ml 3 days before and 7 days after surgery.

Four weeks later, general anesthesia was performed as described above. The K-wires were cut and removed through the former approaches in groups I–III. Animals in group I underwent joint angle measurements as described below and were afterwards euthanized using carbon dioxide (CO_2_) inhalation. Following wound closure, animals of group II and group III were allowed free cage activity for 4 and 8 weeks, respectively, until they underwent joint angle measurements under general anesthesia. Animals in the respective control groups Ic–IIIc were sacrificed after joint angle measurements after 4, 8, and 12 weeks of free cage activity, respectively, without any surgical intervention.

### Joint angle measurement

**Fig. 3:**
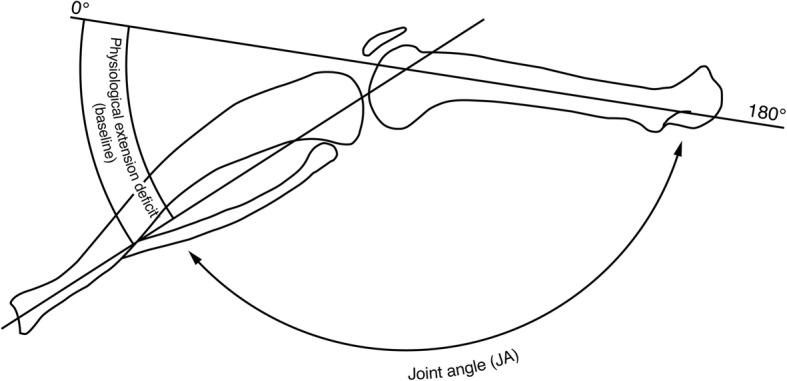
Physiological extension deficit (baseline) in the knee joint of a rat. Graphical illustration of a lateral view of the leg in full extension

The joint angle (JA) was defined as the angle between the longitudinal axis of the femur and the longitudinal axis of the lower leg (line between the tibia plateau and upper ankle joint) in a lateral plain radiograph of the leg. A JA of 180° corresponds to full extension of the knee joint (0°), which is not physiological in rats (Fig. 3).

**Fig. 4:**
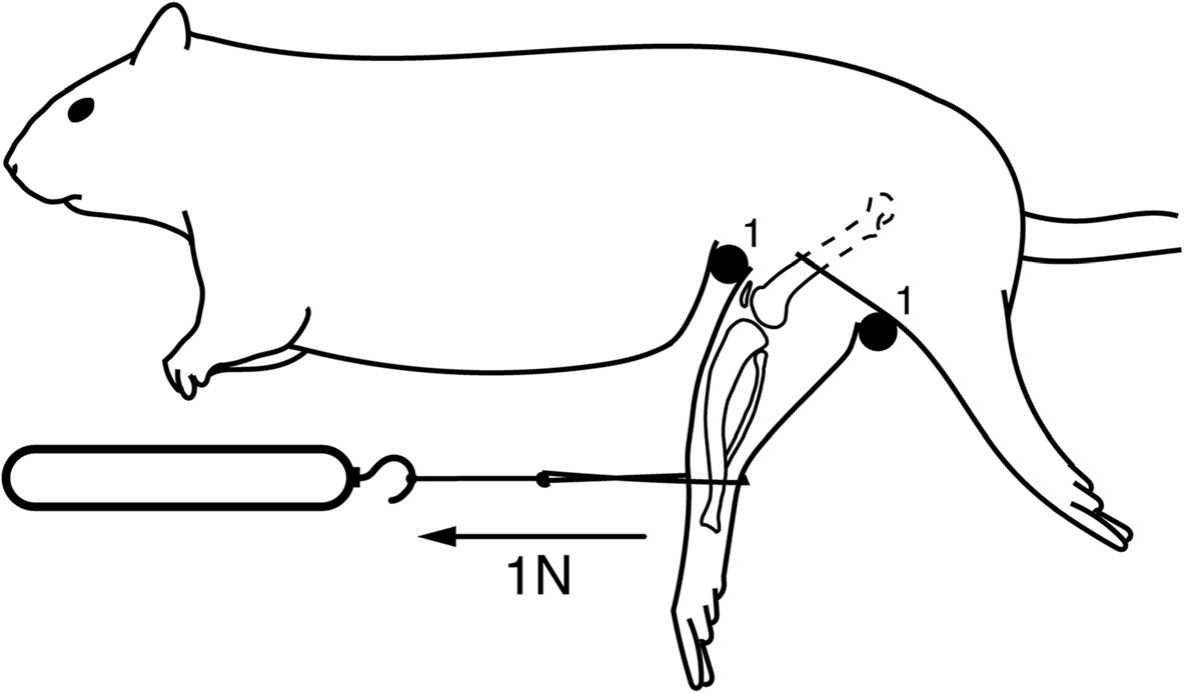
Procedure of joint angle measurement. Graphical illustration of a rat on an acrylic glass rack (permeable to X-rays)

JA measurements were performed in general anesthesia. The animals were placed onto an acrylic glass rack with their operated side below. Plastic pins were used to stabilize the animals’ pelvic and femoral position. A braided cord was attached to the ankle joint, and 35 Nmm of torque was applied using a spring scale (Fig. 4). A torque of 35 Nmm extended the knee joint to its physiological limit but stayed below the level of torque that leads to tearing of the joint capsule (5, 28). Fluoroscopic images were taken using a MX-20 cabinet X-ray system (Faxitron*,* DOM 2009), and joint angles were measured using ImageJ version 1.50e (downloaded from https://imagej.net/) in lateral plain radiographs.

The difference between full extension of 0° and the actual degree of extension in the stretched knee joint (35 Nmm of torque) was specified as extension deficit (ED). Physiological ED (baseline) was determined in the animals of the control group, because a full extension of 0° is not physiological in rats. Joint contracture was defined as the loss of the physiological extension (= mean of ED (treatment group) − mean of ED (control)).

Subsequently, the skin was removed and all soft tissues were circumferentially cut 10 mm proximal and distal to the joint line (periarticular myotomy). Measurements of JA and ED were repeated as described above. After skin removal, extension deficit (ED) is composed of muscular and articular/capsular parts. The gain in extension through periarticular myotomy is consistent with the amount of the myogenic extension deficit (MED). Persisting extension deficit after the myotomy was interpreted as arthrogenic extension deficit (AED).

The loss of the physiological extension by articular structures was interpreted as arthrogenic contracture (AED − AED (controls)). To calculate the amount of the myogenic extension deficit (MED), the extension deficit after myotomy was subtracted from the extension deficit before myotomy (MED = ED − AED). Myogenic joint contracture was defined as the loss of the physiological extension through periarticular muscles (MED − MED (controls)). Extension deficit (ED) is the sum of MED and AED. Animals were sacrificed by inhalation of CO_2_ after completion of the measurements.

To study the anatomic site of lesion, MRI scans were performed with a 3-T MRI system (Magnetom Prisma, Siemens) using a finger coil with a diameter of 4 cm (Siemens) exemplarily in two rat knee joints. Parameters for proton density images were repetition time TR 2910 ms, echo time TE 120 ms, field of view (FOV) 36.00 × 36.00 mm^2^, slice thickness 0.8 mm, and resolution 384 × 384.

### Statistical analysis

Statistical analysis was conducted using the SPSS 24.0 software (SPSS Inc., Chicago, IL, USA). Quantitative results are presented as means ± standard deviation. A Welch-ANOVA with Games-Howell post hoc analysis was carried out. *p* values < 0.05 were considered as statistically significant.

## Results

Two animals died during the initiation of anesthesia and were replaced by other animals. A K-wire dislocation was detected in one rat of group I at the time point of X-ray assessment, so that duration and angle of immobilization were uncertain. Therefore, this animal has been excluded from the study, sacrificed, and not replaced. During the first week after operation, 3 of the 29 operated animals had lost weight. This number reduced to 2, 1, and 1 in the weeks 2, 3, and 4 after operation, respectively. By five postoperative weeks, all animals had regained their initial weight.

**Fig. 5:**
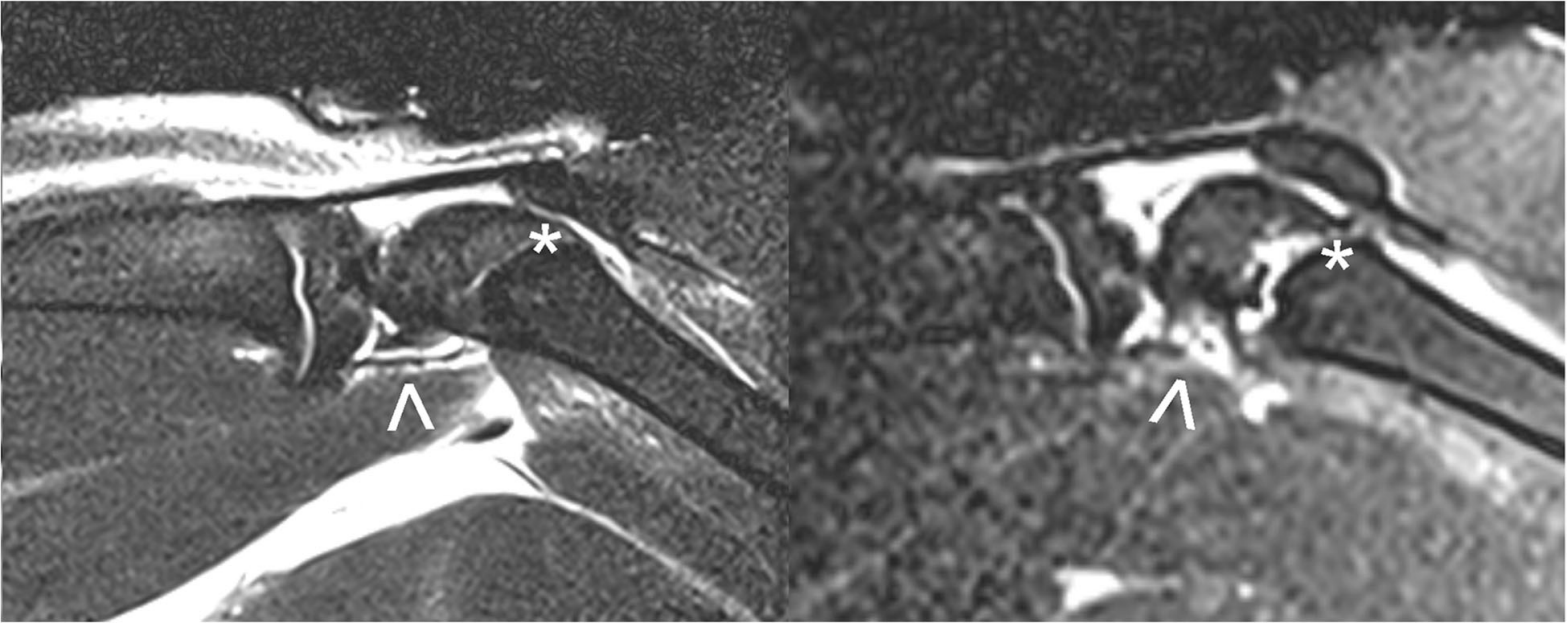
MRI scans of the knee joint. MRI (proton density and T2-weighted turbo spin-echo sequence) scan of the knee joint before (left) and after (right) passive hyperextension of − 45° shows posterior capsular lesion (marked with an arrow) and a posterior widening of the femoral growth plate (anterior part of the growth plate is marked with an asterisk) after the maneuver

In order to delineate the pathomechanism underlying the − 45° hyperextension maneuver of the knee joint, we obtained MRI images of the knee joint exemplarily. We observed a lesion of the posterior capsule with edema and a partial epiphysiolysis with posterior widening of the femoral growth plate (Fig. 5). In fixed flexion, the growth plate was reduced anatomically in all cases (Fig. 2).

Physiological ED (baseline) as determined in the animals of the control group was 43.3° ± 7.4°, 41.5° ± 4.9°, and 43.7 ± 3.2° for groups Ic, IIc, and IIIc, respectively.

The manifestation of the extension deficit (ED) was most pronounced in group I after 4 weeks of immobilization (95.4° ± 15.1°) with a resulting arthrogenic contracture of 55.2°. Four weeks after starting the remobilization (group II), an arthrogenic contracture of 25.7° persisted as compared to the controls, although a significant improvement of extension was noticed (ED 66.6° ± 6.8°, *p* < 0.01). This level remained stable during the further course of remobilization even after 8 weeks (group III, ED 63.5° ± 12.1°, *p* < 0.01 vs. controls, 26.5° arthrogenic contracture) (Table 2). Analysis of the myogenic extension deficit revealed that the flexion contracture was entirely of arthrogenic origin (Table 2).

**Table 2:** Myogenic vs. arthrogenic components of extension deficit (ED). Angles are presented as means ± standard deviation. Contracture is displayed as the difference in extension deficit between intervention group and the respective control

	Extension deficit (ED)	Arthrogenic extension deficit (AED)	Myogenic extension deficit (MED)
Group I (*n* = 9)	95.4° ± 15.1°	74.9° ± 17.0°	20.5° ± 11.9°
Group Ic (*n* = 6)	43.3° ± 7.4°	19.7° ± 3.4°	23.6° ± 8.1
Contracture	52.1°, *p* < 0.01	55.2°, *p* < 0.01	− 3.1°, *p* = 0.99
Group II (*n* = 10)	66.6° ± 6.8°	46.0° ± 12.4°	20.6° ± 14.0
Group IIc (*n* = 6)	41.5° ± 4.9°	20.3° ± 5.6°	21.2° ± 4.8
Contracture	25.1°, *p* < 0.01	25.7°, *p* < 0.01	− 0.6°, *p* = 1.0
Group III (*n* = 10)	63.5° ± 12.1°	50.1° ± 15.4°	13.4° ± 7.7
Group IIIc (*n* = 6)	43.7° ± 3.2°	23.6° ± 7.3°	20.1° ± 7.8
Contracture	17.4°, *p* < 0.01	26.5°, *p* < 0.01	− 6.7°, *p* = 0.64

### Group I and group Ic

**Fig. 6:**
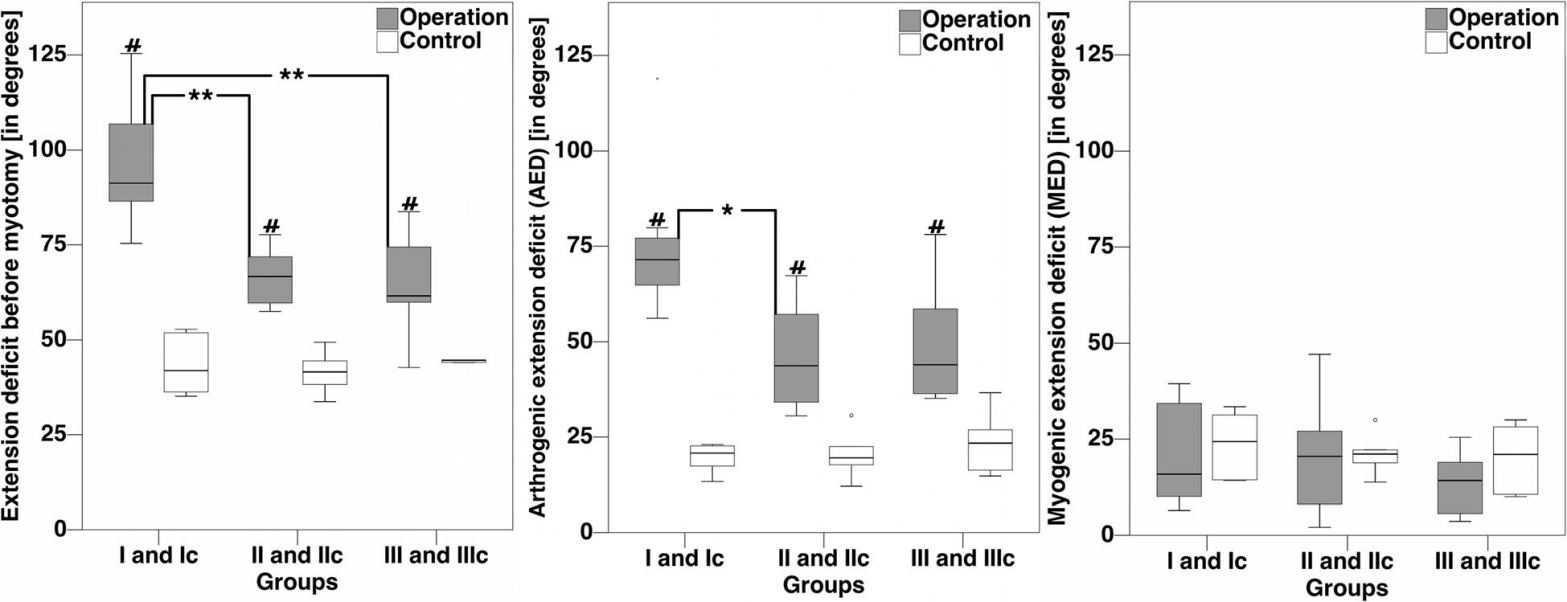
Box plots of extension deficits. Extension deficit (ED, left picture), arthrogenic extension deficit (AED, middle picture), and myogenic extension deficit (MED, right picture) of operated knee joints vs. controls. The difference in extension deficit between an intervention group and its control is defined as contracture. A highly significant difference between an operation group and the respective control is indicated by a hashtag (#*p* < 0.01). A significant difference between operation groups is marked by an asterisk (*p < 0.05, **p < 0.01)

After 4 weeks of immobilization in 145.9° ± 7.5° joint flexion, extension deficit (ED) in group I averaged 95.4° ± 15.1°. Subsequent myotomy increased the extension by 20.5° (MED) and revealed a persisting arthrogenic extension deficit (AED) of 74.9° ± 17.0°. In comparison, the respective control (group Ic, same age, no operation) showed to have an ED of 43.3° ± 7.4°, which was reduced by 23.6° to an AED of 19.7° ± 3.4° via muscular dissection. Differences in ED and AED between the operation group and the control group were statistically significant (*p* < 0.01) (Table 2, Fig. 6). Myogenic extension deficit (MED) did not differ between intervention and control (*p* = 0.99); hence, it did not contribute to the contracture. Therefore, the articular contribution to contracture is 100%. After subtraction of the physiological extension deficit (baseline) of the control, an arthrogenic contracture of 55.2° became evident (*p* < 0.01).

### Group II and group IIc

Four weeks of immobilization in 148.5° ± 8.5° flexion were followed by a 4-week period of remobilization in group II. Although 4 weeks of remobilization lead to an improvement of extension of 28.8° in the mean (group I, ED 95.4° ± 15.1°, group II ED 66.6° ± 6.8°, *p* < 0.01), there was still a noticeable arthrogenic contracture of 25.7°. The differences to the respective control (IIc) were significant (*p* < 0.01) (group IIc, ED 41.5° ± 4.9°, AED 20.3° ± 5.6°). Again, the myogenic component was not different from the control and did not contribute to the contracture (Table 2, Fig. 6).

### Group III and group IIIc

In group III, 4 weeks of immobilization in 145.8° ± 6.8° were followed by a phase of 8 weeks of free remobilization. Even though the first 4 weeks of remobilization have shown to improve the extension deficit (group I vs. group II), contracture level remained stable during the further course of remobilization (arthrogenic contracture group II 25.7°, group III 26.5°). Differences in ED and AED between groups II and III were not significant (*p* = 0.98 and *p* = 0.99), whereas differences between group II and its control were significant (*p* < 0.01 for ED and AED (Table 2, Fig. 6). Arthrogenic components accounted for 100% of the contracture in group III, as MED did not differ from its control (group III, 13.4°, group IIIc 20.1°, *p* = 0.64).

### Comparison between groups I–III

Group I presented a significantly higher ED (95.4° ± 15.1°) than both other groups (group II, 66.6° ± 6.8° and group III, 61.1° ± 14.6°, *p* < 0.01) (Fig. 6). Myotomy lead to a gain in extension of 20.5° and 20.6° in groups I and II, respectively, and to a gain in extension of 13.4° in group III. After myotomy, group I still had the highest loss of extension with an AED of 74.9° ± 17.0° and was significantly different from group II (46.0° ± 12.4°, *p* < 0.05) but not from group III anymore (50.1° ± 15.4°, *p* = 0.06). All groups that underwent an intervention significantly differed in terms of ED and AED from their respective controls, whereas the control groups did not differ significantly among each other (Table 2). When baseline extension deficits of the controls were subtracted, contractures were 55.2°, 25.7°, and 26.5° for groups I, II, and III, respectively. We did not see any difference in ED or AED between group II and group III (*p* = 0.98 and *p* = 0.99 before and after myotomy, respectively Table 2, Fig. 6).

### Composition of contracture

Musculature participated with 20.1°–23.6° in the physiological extension deficit of the control groups, which was not statistically different from the intervention groups with 13.4°–20.6° (Table 2). When MED of the immobilization group is not different from that of the respective control, the myogenic proportion to the contracture can be considered as 0%. Arthrogenic components were responsible for 46.0°–74.9° of the extension deficit in the intervention groups, which was significantly different from the controls. After subtraction of the physiological AED of the respective controls Ic–IIIc, operation groups presented with an arthrogenic contracture of 55.2° in group I and 25.7°–26.5° in group II and group III. Since muscles took no part in the formation of contracture, arthrogenic components were responsible for 100% of the contracture development.

## Discussion

### Extension deficit (ED) and development of contracture

Hildebrand et al. introduced a new model of posttraumatic joint stiffness in the rabbit and used the cortical window to simulate an intraarticular, yet extracartilaginous fracture and to create a hemarthrosis [26]. Nesterenko et al. modified Hildebrand’s model and added a capsular injury, which lead to a stronger contracture than the bony lesion and immobilization alone [21]. There are just a few rat models of PTJS in the knee joint and no one was able to show that the evoked contracture outlasts a period of remobilization. Li et al. for example produced an extension deficit of 124.0° ± 12.3° and a contracture of 95.4° in their traumatic rat model after 8 weeks of knee joint immobilization with polyester sutures, but they never demonstrated that the contracture persisted after remobilization [24]. As previously stated, we observed an ED of 95.4° ± 15.1° before myotomy after only 4 weeks of immobilization. After myotomy, the arthrogenic extension deficit was 74.9° ± 17.0° (arthrogenic contracture of 55.2°). Since Li et al. did not perform a myotomy, the arthrogenic component cannot be assessed in isolation. An arthrogenic contracture of 32° ± 5° was observed by Efird et al., who immobilized the knee joint for 14 days via non-absorbable Ethibond® suture after scraping the trochlear cartilage (Table 3) [28].

**Table 3:** Selection of traumatic small animal models for knee joint contracture

Year	2004	2009	2013	2014
Author	Hildebrand et al.	Nesterenko et al.	Li et al.	Efird et al.
Animal model	Rabbit	Rabbit	Rat	Rat
Bony lesion	Yes	Yes	Yes	–
Hyperextension	–	Yes	Yes	–
Other intervention	–	–	Incision of cruciate ligaments	Cartilage was scraped
Joint immobilization	8 weeks	8 weeks	8 weeks	2 weeks
Fixation type	K-wire	K-wire	Suture	Suture
Remobilization	8, 16, and 32 weeks	16 weeks	–	–
ROM measurement	Yes	Yes	–	–
Main conclusion	New animal model of posttraumatic joint contracture	Hyperextension resulted in additional joint contracture		

In a rat model of traumatic elbow contracture with a 6-week immobilization period in a tubular elastic netting, an AED of 80.2° ± 16° (arthrogenic contracture of 46.6°) after minor and an ED of 100.3° ± 10.8° (arthrogenic contracture of 66.7°) after major capsular damage was observed [22, 23].

Other current rat models did not measure joint angles [25] or did not simulate a posttraumatic joint contracture [7–14]. Some of them measured contracture angles at 4 weeks of immobilization without preceding trauma and reached arthrogenic contractures of 20°–50° [10, 14, 29].

In their traumatic rabbit model with capsular injury, cortical windows, and K-wire fixation for 8 weeks, Nesterenko et al. found an arthrogenic contracture of 76.3° [21].

We were able to produce a joint contracture that is more severe than that of non-traumatic rat models with only 4 weeks of immobilization. Traumatic rat models with a longer period of immobilization or injury of the cartilage are able to produce stronger contractures, when angles are measured at the end of immobilization [24, 28].

### Stability of contracture over time

The arthrogenic contracture induced by minor capsular damage and 6 weeks of immobilization decreased by 27.7° from 46.6° to 18.9° after 6 weeks of remobilization in a rat model of PTJS in the elbow [23]. After major capsular damage, 6 weeks of remobilization reduced the arthrogenic contracture by 45.1° from 66.7° to 21.6° [23]. Accordingly, the arthrogenic contracture in our model decreased by 29.5° from 55.2° to 25.7° after 4 weeks of remobilization and remained at this level (26.5°) after 8 weeks of remobilization. Consequently, the contractures in our model proved to be more stable over time, even if immobilization time was shorter and remobilization longer than those in the latter model. Compared to the control, Trudel et al. found a 37° reduction of arthrogenic contracture from 50° to 13° in their non-traumatic rat model after 4 weeks of immobilization, followed by 4 weeks of remobilization, without further improvement with longer remobilization [10]. In our experiment, we noticed a lower rate of contracture improvement through remobilization.

### Composition of contracture

We differentiated between arthrogenic and myogenic components of the extension deficit (ED). The muscular part of ED did not differ between intervention groups and their respective controls (Table 2, Fig. 6). Thus, we could demonstrate that the posttraumatic contracture originates from the joint and capsule and that there is no additional posttraumatic muscular contribution.

Trudel et al. reported an articular contribution of 56% to knee flexion contracture after 4 weeks of immobilization without preceding trauma in a rat model. This percentage increased to 100% with 4 and 8 weeks of remobilization [6]. In contrast to the latter study, we found an arthrogenic contribution to contracture of 100% after only 4 weeks of immobilization. This leads to the conclusion that our traumatic rat model is more suitable for the creation of an early arthrogenic contracture in comparison to non-traumatic models.

A limitation of our study was that the control group did not undergo a sham operation, which might lead to an overestimation of the influence of capsular disruption and cortical window on joint contracture. Further, assessors of the results were not blinded to the treatment groups, which might lead to subjective bias. We did not study the regain of range of motion after remobilization beyond a twofold duration of immobilization. Data shows that the plateau of contracture is usually reached during the first 8 weeks of recovery [10].

## Conclusions

In this study, we presented a model of stable PTJS in knee joints of rats. Since different structures may be involved in the formation of contractures, we differentiated between muscular and arthrogenic/capsular origins of joint stiffness through angle measurement before and after myotomy of the periarticular muscles. During the weeks of remobilization, the articular range of motion recovered only incompletely and stable joint contractures developed over time with arthrogenic contractures of 26°.

Even if established rabbit models represent a good method, they are expensive and demanding. Due to reduced space requirements and lower costs, rat models have the advantage that they allow for large-scaled testing of therapeutic drugs against joint contracture. This is the reason why drug tests have used especially rat models in recent years [29–31]. Most of the rat models used are either non-traumatic [6–9, 20, 31–33] or did not prove by remobilization, that the evoked contracture is stable [24, 25, 28]. The rat model of PTJS in the elbow published by Lake et al. is promising and complementary to our PTJS model of the rat knee [22]. To our best knowledge, this is the first published rat model of PTJS in the knee joint after major capsular and extracartilaginous bone damage that proved to be stable over time. Future studies with our new model will include medical treatment options for PTJS.

## Abbreviations

AED: Arthrogenic extension deficit
CO_2_: Carbon dioxide
DVSE: German Society of Shoulder and Elbow Surgery
ED: Extension deficit
JA: Joint angle
K-wire: Kirschner wire
MED: Myogenic extension deficit
MFO: Molekulares Forschungszentrum operativer Fächer
MRI: Magnetic resonance imaging
PTJS: Posttraumatic joint stiffness
ROM: Range of motion
